# MicroRNA-302b negatively regulates IL-1β production in response to MSU crystals by targeting IRAK4 and EphA2

**DOI:** 10.1186/s13075-018-1528-9

**Published:** 2018-02-26

**Authors:** Teng Ma, Xiao Liu, Zhifu Cen, Chuan Xin, Mingfeng Guo, Chaoyu Zou, Wenpeng Song, Rou Xie, Kailun Wang, Hong Zhou, Jun Zhang, Zhen Wang, Ce Bian, Kaijun Cui, Jiong Li, Yu-Quan Wei, Jing Li, Xikun Zhou

**Affiliations:** 1 State Key Laboratory of Biotherapy and Cancer Center, West China Hospital, Sichuan University and Collaborative Innovation Center of Biotherapy, Chengdu, 610041 China; 2 Department of Cardiovascular Medicine, West China Hospital, Sichuan University, Chengdu, 610041 China; 3 State Key Laboratory of Oral Diseases, National Clinical Research Center for Oral Diseases, West China College of Stomatology, Sichuan University, Chengdu, 610041 China; 4 Department of Obstetrics and Gynecology, West China Second Hospital, Sichuan University, Chengdu, 610041 China

**Keywords:** MicroRNA-302b, Interleukin-1β, Gouty arthritis

## Abstract

**Background:**

Interleukin-1β (IL-1β) is a pivotal proinflammatory cytokine that is strongly associated with the inflammation of gout. However, the underlying mechanism through which the production of IL-1β is regulated has not been fully elucidated. Our previous work identified that miR-302b had an important immune regulatory role in bacterial lung infections. This study was conducted to evaluate the function of miR-302b on monosodium urate (MSU) crystal-induced inflammation and its mechanism.

**Methods:**

The expression pattern and the immune-regulatory role of miR-302b were evaluated both *in vitro* and *in vivo*. The functional targets of miR-302b were predicted by bioinformatics, and then validated by genetic approaches. In addition, the clinical feature of miR-302b was analyzed using serum samples of patients with gouty arthritis.

**Results:**

The extremely high expression of miR-302b was observed in both macrophages and mouse air membranes treated with MSU. Intriguingly, overexpression of miR-302b regulated NF-κB and caspase-1 signaling, leading to significantly attenuate MSU-induced IL-1β. By genetic analysis, miR-302b exhibited inhibitory function on IRAK4 and EphA2 by binding to their 3′-UTR regions. Corporately silencing IRAK4 and EphA2 largely impaired MSU-induced IL-1β protein production. Moreover, it was also found that miR-302b and EphA2 suppressed the migration of macrophages. Finally, it was observed that high expression of miR-302b was a general feature in patients with gouty arthritis.

**Conclusions:**

These results suggest that miR-302b can regulate IL-1β production in MSU-induced inflammation by targeting NF-κB and caspase-1 signaling, and may be a potential therapeutic target for gouty arthritis.

**Electronic supplementary material:**

The online version of this article (10.1186/s13075-018-1528-9) contains supplementary material, which is available to authorized users.

## Background

Gout is a metabolic disease that is usually characterized by hyperuricemia and the deposition of monosodium urate (MSU) crystals in the joints and subsequent induction of acute inflammatory response and cartilage destruction [1]. The pathogenic process of gout is normally associated with numerous comorbidities, such as chronic kidney diseases, hypertension, obesity, diabetes, and cardiovascular disease [2]. IL-1β is a major effector cytokine and plays a crucial role in the MSU-induced initiation of acute gout flares [1]. Blocking of IL-1β can prevent peritoneal neutrophil accumulation in a mouse model of MSU-induced inflammation, and this appears to be an effective therapy for acute gouty arthritis [3].

The expression and secretion of IL-1β are regulated by several signaling cascades. Highly purified MSU crystals could not solely induce IL-1β production or joint inflammation [4]. The released microbial components (e.g., LPS) during infection and the metabolic compounds of food intake can offer a costimulatory signal that synergizes with MSU crystals to induce IL-1β production. Furthermore, free fatty acids with TLR2/4 are necessary for the induction of IL-1β mRNA and pro-IL-1β [4]. It has been pointed out that the NLRP3 inflammasome is involved in crystal-induced inflammation, and subsequent activation of caspase-1 in the inflammasome can cleave the precursor pro-IL-1β to produce the active IL-1β protein (p17) [5]. However, the underlying mechanism for the regulation of IL-1β production during these processes has not been fully elucidated.

miRNAs are a group of small, endogenous, single-stranded noncoding RNAs that regulate gene expression by mediating messenger RNA (mRNA) cleavage, translation repression, and mRNA destabilization [6]. As tiny regulators of gene expression, miRNAs have been shown to have great potential in cellular processes such as differentiation, apoptosis, and other diverse diseases [7]. However, only a few studies have investigated the role of miRNAs in the pathogenesis of gout [8]. A previous study showed that miR-155 suppressed SH2-containing inositol-5′-phosphatase 1 (SHIP-1) levels and indirectly enhanced the production of proinflammatory cytokines such as tumor necrosis factor alpha (TNF-α) and IL-1β [9]. In another report, overexpression of miR-146a could reduce the expression of IL-1β, TNF-α, monocyte chemoattractant protein-1 (MCP-1), and IL-8 against MSU treatment [10]. However, the individual roles for miRNAs in gout have not been fully elucidated.

Our previous study revealed that miR-302b is a novel inflammatory regulator of TLR/NF-κB signaling in respiratory bacterial infections [11]. To find out whether miR-302b regulates proinflammatory cytokines in the pathogenesis of gout, in the present study with bioinformatics and genetic approaches we defined that miR-302b fine-tuned IL-1β production by targeting the NF-κB pathway. It was further demonstrated that IRAK4 and EphA2 were the functional targets of miR-302b, and either enhancement of miR-302b or silence of EphA2 suppressed the migration of macrophages. These findings suggest that miR-302b plays an important role in the pathogenesis of MSU crystal-induced inflammation.

## Methods

### Cells

THP-1 cells from American Type Culture Collection (Manassas, VA, USA) were cultured in RPMI 1640 medium supplemented with 10% heat-inactivated FBS and penicillin–streptomycin. THP-1 cells were exposed to 100 ng/ml phorbol myristate acetate (PMA; Beyotime Biotechnology, Shanghai, China) on the day before the indicated experiment.

### Mice

Eight-week-old male BALB/c mice were purchased from the Dossy Experimental Animals Company (Chengdu, China). Animals were housed in a specific pathogen-free facility of the State Key Laboratory of Biotherapy, Sichuan University, Chengdu, China. All animal studies were approved by the Ethics Committee of the State Key Laboratory of Biotherapy, Sichuan University.

### Clinical serum sample collection

A total of 38 human serum samples were obtained from 18 patients diagnosed with acute gouty arthritis during an acute gout flare, and 20 healthy subjects were randomly selected from healthy individuals who participated in a physical examination at the West China Hospital from June 2017 and July 2017 (Additional file 1: Table S1). Once serum samples were collected, they were stored immediately at −80 °C. Written informed consents were provided by all participants, and the study was approved by the Ethics Committee of the West China Hospital, Sichuan University.

### Reagents

MSU crystals were purchased from InvivoGen (San Diego, CA, USA) and used freshly for in-vitro treatment by dissolving in phosphate buffered saline (PBS). Antibodies for cleaved caspase-1 (p20), phospho-NF-κB p65 (ser536), and β-actin were purchased from Cell Signaling Technology (Danvers, MA, USA). Human and mouse mature IL-1β were measured using the IL-1β ELISA kit (Neobioscience, Shenzhen, China). PerCP-CD45, PE-F4/80, and APC-Gr-1 antibodies were obtained from BioLegend (San Diego, CA, USA).

### Transfection

THP-1 cells were seeded in six-well or 24-well plates with 100 ng/ml PMA overnight. After washing the cells once with PBS, they were transfected with negative control miRNA mimics (NS-m), miR-302b mimics (302b-m), negative control siRNA (si-NC), IRAK4 siRNA (si-IRAK4), or EphA2 siRNA (si-EphA2) purchased from RiboBio Co., Ltd (Guangzhou, China) using Lipofectamine® 3000 (Life Technologies, Grand Island, NY, USA).

### Measurement of miRNAs and mRNA expression

Total RNA was isolated from samples with TRIzol™ Reagent (Life Technologies) and dissolved in RNase-free water. For human serum samples, *Caenorhabditis elegans* miRNA cel-miR-39 was added as a synthetic spike-in control RNA. For mature miRNA detection, reverse transcription was performed using a miScript II RT Kit (Qiagen, Valencia, CA, USA), and real-time qPCR was performed using a miScript SYBR® Green PCR Kit (Qiagen). For mRNA detection, reverse transcription was performed using a PrimeScript™ RT reagent Kit with gDNA Eraser (Takara, Dalian, China), and real-time qPCR was performed using SYBR® Premix Ex Taq™ II (Takara). The miRNA and mRNA detections were performed on a CFX96 Touch™ Real-Time PCR Detection System (Bio-Rad, Hercules, CA, USA), and the data were analyzed with CFX Manager™ software version 3.1 (Bio-Rad). The levels of mature miRNA were normalized against the control U6 snRNA (human source cell samples), sno202 (mouse source cell samples), or cel-miR-39 (human source serum samples). The levels of EphA2 and IRAK4 were normalized against GAPDH. The primers used in this study are presented in Additional file 1: Table S2.

### Luciferase assay

THP-1 cells were cotransfected with miRNA (NS-m or 302b-m) and the luciferase reporter vector containing wild-type or point-mutated 3′ UTR (WT UTR or mutant UTR) of IRAK4 and EphA2 using Lipofectamine® 3000 (Life Technologies). Luciferase expression levels were measured at 24 h post transfection using a dual-luciferase reporter assay system according to the manufacturer’s instructions (Promega, Madison, WI, USA).

### Western blot analysis

The antibodies for β-actin (1:1000), phosphor-NF-κB p65 (1:1000), and cleaved caspase-1 p20 (1:1000) were used for western blot analysis. The quantitative analysis for the results of the western blot analysis was performed using the Gel-Pro analyzer 4.0 (Media Cybernetics, Bethesda, MD, USA).

### *In-vitro* migration assay

A Boyden chamber with an 8-μm porous membrane (Corning) in the 24-well plate was used for the migration assay. Briefly, THP-1 cells were transfected with NS-m, 302b-m, si-NC, or si-EphA2 for 48 h. The cell numbers were counted with a hemocytometer and resuspended with RPMI 1640 medium without serum. Then 500 μl cell suspension containing the indicated cell number was loaded into the Boyden chamber, whereas 1 ml RPMI 1640 medium with 5% serum was placed in the bottom compartment. After incubating at 37 °C for 24 h, cells on the upper side of membranes were removed. The migratory cells on the lower side of the membrane were stained with crystal violet and then counted under light microscope.

### Confocal microscopy

THP-1 cells were transfected with NS-m or 302b-m and si-EphA2 or NC respectively for 48 h, and then the cells were treated with MSU for another 1 h. The cells were fixed with 4% paraformaldehyde and permeabilized with 0.3% Triton-X 100. Rhodamine phalloidin plus DAPI were diluted into PBS, and the cells were incubated at room temperature in the dark for 30 min. Rhodamine phalloidin-labeled F-actin (red) and DAPI-labeled nuclei (blue) were detected using confocal microscopy (Nikon TI-DH, Japan).

### Mouse air pouch model

The backs of mice (four to seven mice per group) were subcutaneously injected with 2 ml sterile air and followed by a second injection of 3 ml sterile air after 3 days. The miR-302b agomir (302b-a) and negative control (NS-a) were injected into the air pouches on days 2 and 4. At 6 days after the first injection, 2 mg of MSU crystals in 0.5 ml of PBS or 0.5 ml of PBS alone were injected into the air pouches. After 6 h, the mice were anesthetized, and the air pouch fluids were lavaged with 3 ml of PBS. The lavages were centrifuged at 1000 × *g* for 5 min. The cell pellets were stained with CD45, Gr-1, and F4/80 antibodies for flow cytometry analysis, and the supernatants were used for ELISA. For immunoblot assays, air pouch lavages were precipitated to obtain protein pellets. For histological analysis, sagittal sections of air pouches were fixed in 10% paraformaldehyde and stained with hematoxylin and eosin (H&E).

### Statistical analysis

All statistical analyses were conducted with SPSS 21.0 software. Data are presented as the mean ± SDEVs or SEMs. Statistical analysis was performed using Student’s *t* test for comparing two groups. Differences in the mean values were considered to be significant at *p* < 0.05.

## Results

### miR-302b expression was upregulated after MSU treatment

**Fig. 1:**
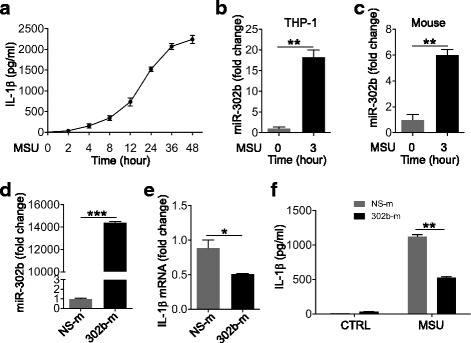
miR-302b expression altered after MSU treatment and repressed MSU-induced IL-1β expression *in vitro*. **a** Time-dependent IL-1β production in THP-1 cells. THP-1 cells treated with 150 μg/ml MSU and cell supernatant collected at a different time points from 2 to 48 h. IL-1β protein levels in THP-1 cells detected with ELISA. **b** THP-1 cells treated with MSU for 1 h and 12 h. Cell lysates analyzed for miR-302b expression with real-time qPCR. **c** Air pouch inflammation model made with an injection of sterile air. Six hours after injection of MSU crystals, three mice were anesthetized and air pouch membranes collected. Expression of miR-302b detected using real-time qPCR. **d** Real-time qPCR analysis of miR-302b expression in THP-1 cells transfected with miRNA negative control (NS-m) or miR-302b mimics (302b-m). **e** Real-time qPCR analysis of IL-1β mRNA levels in miRNA NS-m or 302b-m-transfected THP-1 cells after treatment with MSU. **f** ELISA analysis of IL-1β protein levels in cell culture medium 8 h after MSU treatment. THP-1 cells transfected as indicated in (**e**). Data represent three experiments, shown as means ± SEMs (***p < 0.001, **p < 0.01, *p < 0.05 by *t*-test). IL-1β interleukin-1β, MSU monosodium urate, CTRL control

PMA-induced differentiation of THP-1 monocytes into macrophages was used as an *in-vitro* experimental model. Since IL-1β is an indicator of gout inflammation [12], we first explored a rational MSU treatment for subsequent assays by detecting the expression of IL-1β. The PMA-induced THP-1 cells were treated with 150 μg/ml MSU at different time points from 0 to 48 h. The results showed that the IL-1β protein level was increased in a time-dependent manner upon MSU stimulation (Fig. 1a). Then we analyzed miR-302b expression in THP-1 cells with/without MSU treatment, showing that miR-302b expression was significantly upregulated at 3 h post MSU treatment (Fig. 1b; Additional file 1: Table S2). Based on the established mice air pouch model, the air membranes were dissected to detect the possible alteration for the miR-302b expression. As shown in Fig. 1c, the expression of miR-302b in mouse air membranes was also significantly increased in the MSU-treated group, suggesting that miR-302b might be involved in MSU-induced gouty arthritis.

### miR-302b attenuated the MSU-induced inflammatory response both *in vitro* and *in vivo*

To determine the potential role of miR-302b in MSU treatment, we examined the effect of miR-302b on MSU-induced inflammatory cytokine gene expression in THP-1 cells. We first showed by qRT-PCR that the chemically synthesized miRNA mimics (302b-m) could strikingly elevate the expression level of miR-302b (Fig. 1d) and inhibit MSU-induced IL-1β mRNA expression compared to a miRNA mimic negative control (NS-m) (Fig. 1e). Consistent with this result, ELISA was able to show that the protein level of IL-1β in cells overexpressing miR-302b was also lower compared to the cells transfected with the NS-m (Fig. 1f).

**Fig. 2:**
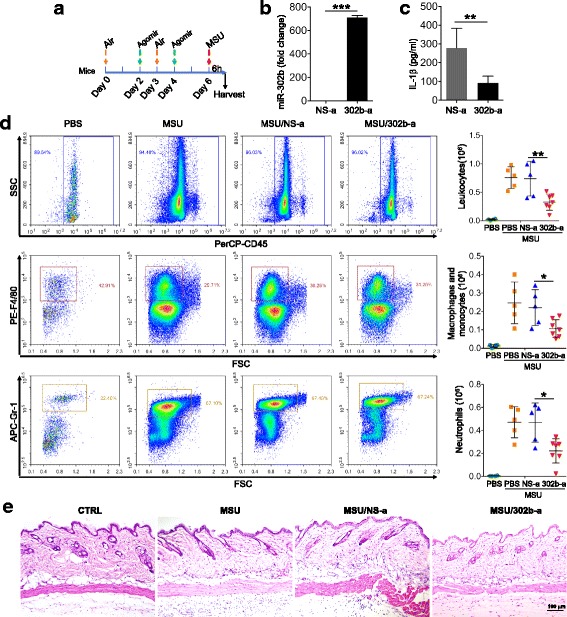
miR-302b repressed MSU-induced inflammatory response *in vivo*. Four to seven mice injected subcutaneously with 2 ml of sterile air followed by a second injection of 3 ml of sterile air after 3 days. miR-302b agomir (302b-a) and a negative control (NS-a) injected into air pouches on days 2 and 4. Six days after the first injection, 2 mg of MSU crystals in 0.5 ml of PBS or 0.5 ml of PBS alone injected into air pouches. After 9 h, air pouch fluid harvested by injecting 3 ml PBS. **a** Schematic of *in-vivo* air pouch model. **b** Air pouch membranes dissected and expression of miR-302b detected by QPCR. **c** Supernatants of air pouch fluid analyzed by ELISA for IL-1β. **d** After centrifuging, precipitated cells from air pouch fluid were stained with PerCP-CD45 Ab, PE-F4/80 Ab, and APC-Gr-1 Ab. Percentage and cell number of migrated leukocytes, microphages, and neutrophils analyzed using flow cytometry. **e** Sagittal sections of air pouches fixed in 4% paraformaldehyde and stained with hematoxylin and eosin (H&E). Data shown as means ± SEMs (**b, c**) or means ± SDEVs (**d**) (***p < 0.001, **p < 0.01, *p < 0.05 by *t* test). IL-1β interleukin-1β, PBS phosphate buffered saline, MSU monosodium urate, CTRL control, SSC side scatter, FSC forward scatter

Given the correlation between miR-302b and IL-1β expression, we next sought to investigate the role of miR-30b in a widely used MSU-induced mouse air pouch inflammatory model [13–15]. miR-302b agomir (302b-a) or a negative control miRNA agomir (NS-a) was reconstituted with PBS and air pouch administration (1 nmol/mouse/per dose) 2 and 4 days prior to MSU treatment (Fig. 2a). miR-302b expression was first examined in mouse air pouch membrane tissues. Two doses after 302b-a injection, miR-302b was found to be increased more than 700 times in the presence of NS-a (Fig. 2b). As expected, the IL-1β expression level in air pouch fluid was reduced in the 302b-a group compared with that of NS-a after MSU stimulation (Fig. 2c). Furthermore, it has been reported that MSU could induce macrophage and neutrophil infiltration into the air pouch [16]. To further verify the relationship between the reported phenomenon and IL-1β expression, we next assessed the number of macrophages and neutrophils infiltrating the air pouch by staining the cells with macrophage marker F4/80 and neutrophil marker Gr-1. As shown in Fig. 2d, MSU enhanced the infiltration of macrophage and neutrophils into the air pouch, but this was dramatically inhibited by 302b-a. Notably, there was no difference between the ratio of infiltrating macrophages and neutrophils in the 302b-a and NS-a groups (Fig. 2d). In addition, 302b-a also attenuated leukocyte infiltration in the air pouch tissues induced by MSU, as shown by the histological examination (Fig. 2e). Thus, these findings suggest that miR-302b played a negative regulatory role in regulating the expression of MSU-induced inflammatory cytokines in gout.

### miR-302b inhibited NF-κB and caspase-1 signaling

**Fig. 3:**
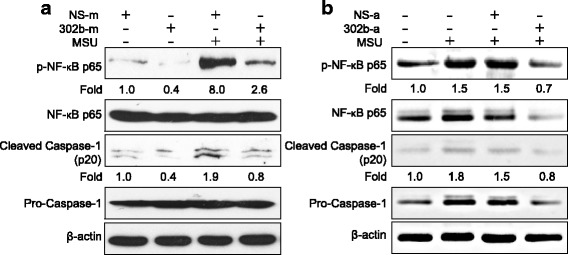
miR-302b inhibited NF-κB and caspase-1 signaling. **a** THP-1 cells transfected with NS-m or 302b-m for 48 h. Six hours after MSU treatment, phosphorylation level of NF-κB and cleaved caspase-1 p20 detected by western blot analysis. **b** Air pouch membranes dissected from the mice used for Fig. 2 analyzed by immunoblotting for NF-κB and caspase-1 (p20). Densitometric quantification of western blotting gel shown as fold change under the band. Data represent three experiments. MSU monosodium urate, NS-m negative control mimic, 302b-m miR-302b mimic, 302b-a miR-302b agomir, NS-a negative control agomir

These results have shown that miR-302b can reduce MSU-induced IL-1β expression at both mRNA and protein levels, indicating that miR-302b may be involved in the transcriptional and maturation process of IL-1β. It is well known that TLR/NF-κB signaling regulates the transcriptional activity of IL-1β, and MSU-induced NLRP3/Caspase-1 activation is involved in the maturation of IL-1β [5, 11]. We next elucidated the direct effects of miR-302b on the activation of NF-κB and caspase-1 during MSU stimulation. Subsequently, the effect of miR-302b on phosphorylation of the NF-κB p65 subunit in THP-1 cells was measured, indicating that the phosphorylation of NF-κB p65 subunit was majorly eliminated in 302b-m-transfected THP-1 cells when compared with the cells transfected with NS-m (Fig. 3a). Active caspase-1 heterodimer is formed by autoproteolysis-mediated pro-caspase-1 activation following inflammasome assembly, and this cleavage is the hallmark of inflammasome activation, which is required for pyroptosis and IL-1β secretion [17]. In our setting, we found that the level of cleaved caspase-1 p20 subunit was suppressed by miR-302b, thereby indicating the identical tendency between the cleaved caspase-1 p20 and the cleavage of precursor IL-1β-produced mature IL-1β (Fig. 3a).

To determine whether miR-302b has any effect on NF-κB signaling and inflammasome activation *in vivo*, we then dissected the air pouch membranes used for Fig. 2 to detect the phosphorylation of the NF-κB p65 subunit and the cleaved caspase-1 p20 subunit. As shown in Fig. 3b, enforced expression of miR-302b via agomir in air pouch membranes reduced the endogenous level of the phosphorylation of NF-κB p65 and cleaved caspase-1 p20 (Fig. 3b). These data suggest that the inhibitory role of miR-302b in MSU-induced proinflammatory gene expression is due to its effects on the TLR signaling pathway from downregulating the activation of NF-κB and the NLRP3 inflammasome signaling pathway by impairing the activation of capase-1.

### IRAK4 and EphA2 were the functional targets of miR-302b

**Fig. 4:**
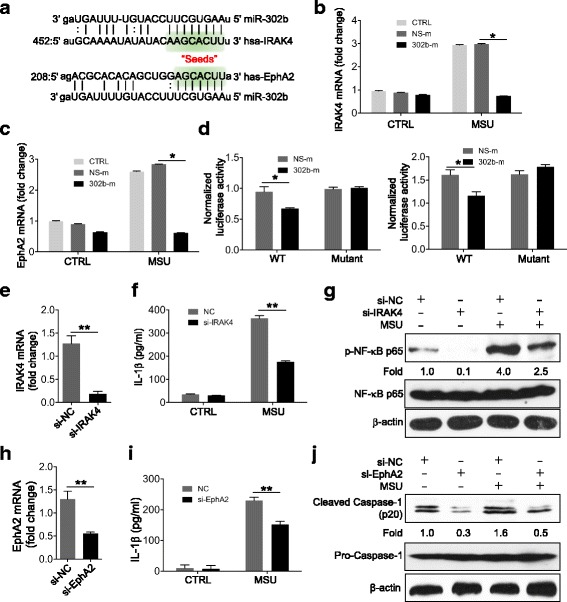
IRAK4 and EphA2 were functional targets of miR-302b. **a** IRAK4 3′ UTRs and EphA2 3′ UTRs contain one predicted miR-302b binding. Predicted duplex formations between IRAK4 3′ UTR and miR-302b (upper), and EphA2 3′ UTR and miR-302b (lower). **b, c** Real-time qPCR analysis of IRAK4 and EphA2 mRNA levels in NS-m and 302b-m-transfected THP-1 cells with/without treatment of MSU. **d** Normalized luciferase activity of a reporter containing wild-type or point-mutated 3′ UTR reporter constructs (WT UTR or mutant UTR) of IRAK4 (left) and EphA2 (right) in THP-1 cells cotransfected with NS-m or 302b-m. **e–j** THP-1 cells transfected with a negative control siRNA (si-NC), IRAK4 siRNA (si-IRAK4), or EphA2 siRNA (si-EphA2) for 48 h. **e** Real-time qPCR analysis of IRAK4 mRNA level in THP-1 cells transfected with negative control siRNA si-NC or si-IRAK4. **f** si-NC or si-IRAK4-transfected THP-1 cells treated with MSU for 8 h. ELISA of IL-1β protein levels in cell culture medium. **g** Western blot analysis of p-NF-κB in THP-1 cells treated with MSU treatment for 3 h after transfection with si-NC or si-IRAK4. **h** Real-time qPCR analysis of EphA2 mRNA level in THP-1 cells transfected with negative control siRNA si-NC or si-EphA2. **i** si-NC or si-EphA2-transfected THP-1 cells treated with MSU for 8 h. ELISA of IL-1β protein levels in cell culture medium. **j** Western blot analysis of cleaved caspase-1 p20 in THP-1 cells treated with MSU treatment for 3 h after transfection with si-NC or si-EphA2. Densitometric quantification of western blotting gel shown as fold change under the band. Data represent three experiments, shown as means ± SEMs (**p < 0.01, *p < 0.05 by *t* test). IRAK-4 interleukin-1 receptor-associated kinase 4, EphA2 EPH receptor A2, CTRL control, MSU monosodium urate, NS-m negative control mimic, 302b-m miR-302b mimic, IL-1β interleukin-1β

We further used bioinformatics tools, such as miRDB, TargetScan, and miRanda, to predict the molecular targets of miR-302b. Then we paid attention to the recurrent genes associated with inflammation in all three applications, and interleukin-1 receptor-associated kinase 4 (IRAK4) and Eph receptor A2 (EphA2) were the hot targets of miR-302b. The three-prime untranslated region (3′-UTR) of IRAK4 and EphA2 was predicted to be bound by the “seed sequence” of miR-302b (Fig. 4a). IRAK4 is an important upstream molecule of the NF-κB signaling pathway and has been shown to regulate proinflammatory gene expression in a bacterial infection model [11]. Additionally, EphA2 is a member of the Eph receptor tyrosine kinase (RTK) family and regulates inflammation and immune cell trafficking [18, 19]. RT-qPCR results were firstly used to confirm that IRAK4 and EphA2 were the targets of miR-302b target (Fig. 4b, c). We further determined that IRAK4 and EphA2 expression was enhanced upon MSU treatment, found in MSU-stimulated THP-1 cells (Fig. 4b, c), and these results indicated that IRAK4 and EphA2 may function in MSU-induced inflammation. To further confirm the target genes of miR-302b, luciferase constructs containing mutated 3′ UTR of IRAK4 and EphA2 were used in the luciferase reporter gene experiments. As shown in Fig. 4d, overexpression of miR-302b inhibited the activity of a luciferase reporter construct containing IRAK4 3′ UTR and EphA2 3′ UTR, but not the mutated 3′ UTR of IRAK4 and EphA2 (Fig. 4d).

We subsequently evaluated whether IRAK4 and EphA2 were the functional targets of miR-302b by siRNA-mediated IRAK4 and EphA2 knockdown in the THP-1 cells (Fig. 4e, h), and ELISA results showed that the IL-1β protein expression level was repressed in the supernatant of THP-1 cells transfected with IRAK4 or EphA2 siRNA compared to the controls (Fig. 4f, i). IRAK4 has been shown to play a critical role in TLR/NF-κB-dependent immune responses by inducing NF-κB phosphorylation [20]. Our western blot analysis showed that the phosphorylation level of the NF-κB p65 subunit in THP-1 cells transfected with IRAK4 siRNA (si-IRAK4) was significantly decreased compared to the cells transfected with control siRNA (si-NC) (Fig. 4g). However, there was no significant change in the cleavage of the caspase-1 p20 subunit level after IRAK4 silencing (Additional file 1: Figure S1a). Silencing EphA2 in THP-1 cells resulted in decreased cleavage of the caspase-1 p20 subunit, but did not affect the phosphorylation level of NF-κB p65 subunit, compared to si-NC transfected cells (Fig. 4j, Additional file 1: Figure S1b). Collectively, these data strongly indicated that miR-302b inhibits the NF-κB signaling-mediated IL-1β mRNA transcription by directly targeting IRAK4, and inhibits caspase-1-mediated IL-1β protein maturation by directly targeting EphA2. Thus, miR-302b regulated the production of mature IL-1β in the two aspects.

Additionally, MSU phagocytosed by resident macrophages resulted in the production of inflammatory cytokines and chemokines, including TNF-α [21]. Indeed, our data also showed the upregulated expression of TNF-α in macrophages after exposure to MSU. The expression level of TNF-α could be downregulated by miR-302b (Additional file 1: Figure S2a). However, these two targets, IRAK4 and EphA2, had no effect on the regulation of TNF-α expression (Additional file 1: Figure S2b, c).

### Overexpressing miR-302b and silencing EphA2 inhibited cell migration

**Fig. 5:**
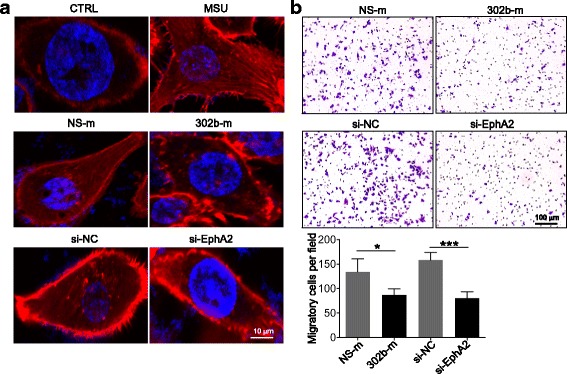
Overexpression of miR-302b and interference with EphA2 could inhibit cell migration. THP-1 cells transfected with NS-m, 302b-m, si-NC, or si-EphA2 for 48 h. **a** THP-1 cells subsequently treated with MSU for another 1 h. Rhodamine phalloidin-labeled F-actin (red) and DAPI-labeled nucleus (blue) analyzed by confocal microscopy. **b** Transwell assay for THP-1 cells plated on upper cell culture inserts. Data represent three experiments, shown as means ± SEMs (***p < 0.001, *p < 0.05 by *t* test). CTRL control, MSU monosodium urate, NS-m negative control mimic, 302b-m miR-302b mimic, si-NC negative control siRNA, si-EphA2 EPH receptor A2 siRNA

Our results suggested that miR-302b attenuated macrophage and neutrophil infiltration (Fig. 2), and previous studies reported that EphA2 could regulate the expression of cytokines and leukocyte infiltration in a mouse model [22–24]. To determine whether miR-302b and EphA2 influence cell migration, we next investigated the effects of miR-302b and EphA2 on the migration ability of THP-1 cells. Filament actin (F-actin) plays a very important role in cell movement and migration, and the formation of F-actin is a marker of cell transformation [25]. Phalloidin staining results showed that the formation of F-actin was largely suppressed in 302b-m or si-EphA2-transfected THP-1 cells with MSU treatment (Fig. 5a). Meanwhile, we determined THP-1 cell migration by staining the nuclei of the migratory cells on the underside of the inserted Transwell membrane. As expected, THP-1 cells transfected with NS-m or si-NC had markedly high migration ability, and the migrated cell number of miR-302b or si-EphA2 transfection decreased approximately 30% and 50%, respectively (Fig. 5b). These results suggested that miR-302b and EphA2 may be involved in the recruitment of inflammatory cells into the inflammatory site by regulating the migration of cells during the MSU-induced inflammatory response in gouty arthritis.

### miR-302b expression level was high in gouty arthritis patients

**Fig. 6:**
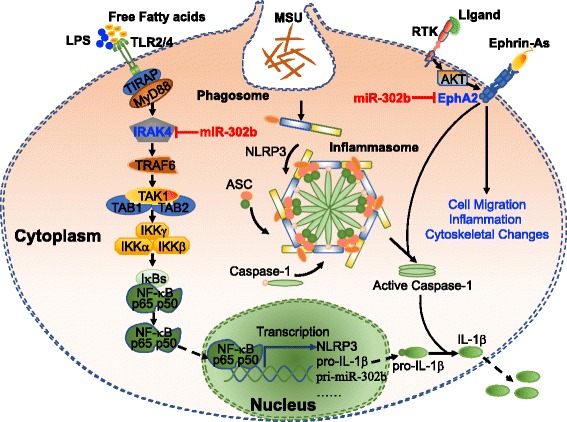
Schematic model for critical role of miR-302b in MSU-induced inflammation. Purified MSU crystals cannot induce IL-1β alone and a second stimulus is needed. Food intake, alcohol consumption, or microbial components released during infection could synergize with MSU crystals to induce release of IL-1β and enhance inflammation. miR-302b works as a negative regulator in TLR and caspase-1 signaling by targeting IRAK4 and EphA2. Furthermore, overexpression of miR-302b and interference with EphA2 could also inhibit cell migration. MSU monosodium urate, IL-1β interleukin-1β, IRAK-4 interleukin-1 receptor-associated kinase 4, EphA2 EPH receptor A2, LPS lipopolysaccharide, TLR Toll-like receptor, RTK receptor tyrosine kinase

To examine the prognostic value of circulating miR-302b in gouty arthritis (GA), we finally determined the levels of miR-302b in the serum of GA patients during an acute gout flare. The characteristics of healthy controls and GA patients are summarized in Additional file 1: Table S1. As shown in Additional file 1: Figure S3, the levels of circulating miR-302b were changed significantly in GA patients compared to controls. These results indicated that the elevated expression of miR-302b was obviously associated with GA patients. Collectively, our findings implicate that miR-302b plays a crucial role in the development of gout flares and represents a novel strategy to prevent excessive inflammation in gouty arthritis (Fig. 6).

## Discussion

In the past few years, an increasing number of miRNAs were found to be associated with the regulation of inflammation [26, 27]. Several studies have shown that many miRNAs, such as miR-155 and miR-451, were dysregulated in the synovial membrane and regulated the inflammatory response in clinical or experimental arthritis [28–31]. These data indicated that miRNAs play important roles in the physiological and pathological processes of arthritis. The miR-302/367 family is involved in multiple kinds of diseases, including tumors, immune diseases, cardiovascular diseases, and so forth [32–34]. miR-302b is a member of the miR-302/367 family. Researchers have shown that miR-302b can suppress tumor proliferation of esophageal squamous cell carcinoma (ESCC) via downregulation of the Erb-b2 receptor tyrosine kinase 4 (*ERBB4*) gene [35]. In our previous study, miR-302b could attenuate bacteria-induced inflammatory responses via a negative feedback for TLR signaling [11]. In this study, we found that the expression of miR-302b was upregulated in THP-1 cells and mouse tissues after MSU treatment, as well as in the serum of GA patients. This finding suggested that miR-302b plays an important role in gouty arthritis.

Immune cells, especially local macrophages and their secretion of cytokines, play a very important role in the pathogenesis of gouty arthritis [36]. IL-1β and TNF-α are thought to be important proinflammatory factors leading to acute and chronic gout arthritis in patients, whereas IL-1β plays a vital role in the development of GA [12]. Dalbeth *et al.* [10] found that miR-146a was increased in intermittent episodes of gout, and overexpression of miR-146a could downregulate IL-1β, TNF-α, and IL-8 at protein level in MSU-induced acute inflammatory responses. In line with the study, we also found that overexpression of miR-302b suppressed TNF-α protein production, but the TNF-α protein level was not affected by the target genes of miR-302b, IRAK4, and EphA2 (Additional file 1: Figure S2). These findings suggested that miR-302b regulates the expression of TNF-α by targeting gene(s) beyond IRAK4 and EphA2.

Previous studies demonstrated that EphA2 regulated the migration of cells by promoting the activation of RhoA protein [37]. Our results showed that silencing the EphA2 gene could affect F-actin formation and subsequently inhibit cell migration. Then we asked ourselves whether EphA2 regulates the migration of cells by affecting RhoA or other proteins during MSU stimulation. To this end, we firstly defined that EphA2 can regulate the cleavage of caspase-1 in the NLRP3 inflammasome, which may have an important influence on the pathogenesis of GA. However, the molecular mechanism needs to be studied in the future.

## Conclusions

Taken together with all of the data for the function of miR-302b in MSU-induced inflammation and its mechanism, we believe that miR-302b could regulate the transcription and maturation of IL-1β by targeting IRAK4 and EphA2, respectively. Additionally, IRAK4 and EphA2 gene expression could downregulate MSU-induced IL-1β protein production. Based on these findings, miR-302b is likely an important negative regulator in the inflammation of gouty arthritis, and may have great potential to serve as a therapeutic target in gouty arthritis.

## Additional file

Additional file 1: Table S1. presenting patient characteristics, **Table S2.** presenting primers used in this study, **Figure S1.** showing function of IRAK4 and EphA2 on level of cleaved caspase-1 p20 and p-NF-κB in THP-1 cells, respectively, **Figure S2.** showing miR-302b represses MSU-induced IL-1β expression, and **Figure S3.** showing miR-302b serum levels in gouty arthritis patients and healthy controls. (DOCX 1301 kb)

## Abbreviations

302b-a: miR-302b agomir
302b-m: miR-302b mimic
3′ UTR: Three-prime untranslated region
EphA2: EPH receptor A2
IL-1β: Interleukin-1β
IRAK-4: Interleukin-1 receptor-associated kinase 4
MCP-1: Monocyte chemoattractant protein-1
MSU: Monosodium urate
NS-a: Negative control agomir
NS-m: Negative control mimic
PMA: Phorbol myristate acetate
SHIP-1: SH2-containing inositol-5′-phosphatase 1
si-EphA2: EphA2 siRNA
si-IRAK4: IRAK4 siRNA
si-NC: Negative control siRNA
TNF-α: Tumor necrosis factor alpha
