# Supplementary material for: MicroRNA-302b negatively regulates IL-1β production in response to MSU crystals by targeting IRAK4 and EphA2
Source: Arthritis Res Ther. 2018 Feb 26;20:34. doi: 10.1186/s13075-018-1528-9 (PMC5828083; doi:10.1186/s13075-018-1528-9)
Supplement: Supplementary file 1 — presenting patient characteristics, Table S2. presenting primers used in this study, Figure S1. showing function of IRAK4 and EphA2 on level of cleaved caspase-1 p20 and p-NF-κB in THP-1 cells, respectively, Figure S2. showing miR-302b represses MSU-induced IL-1β expression, and Figure S3. showing miR-302b serum levels in gouty arthritis patients and healthy controls. (DOCX 1301 kb) [file 13075_2018_1528_MOESM1_ESM.docx]

MicroRNA-302b negatively regulates IL-1β production in response to MSU crystals by targeting IRAK4 and EphA2

# Supplementary Tables

**Table S1: Patient characteristics.**

| Characteristic | Healthy Control Group  (n = 20) | Gouty Arthritis Group  (n = 18) |
| --- | --- | --- |
| Mean age in years (range) | 41.765 (20-64) | 40.25 (27-54) |
| Sex | Male | Male |
| T-test | 0.655 |  |

**Table S2: The primers used in this study.**

| Name | Sequence (5' to 3') |
| --- | --- |
| miR-302b-F | GCGGCGTAAGTGCTTCCATGTTTTA |
| snoRNA202-F | GCCTTTTGAACCCTTTTCCATCTG |
| U6-F | CTCGCTTCGGCAGCACATATACT |
| U6-R | ACGCTTCACGAATTTGCGTGTC |
| cel-miR-39 | CCTCACCGGGTGTAAATCAG |
| GAPDH-F | TCTCGCTCCTGGAAGATGGT |
| GAPDH-F | GGAAGGTGAAGGTCGGAGTC |
| IL-1β-F | CAGAAGTACCTGAGCTCGCC |
| IL-1β-R | CATGGCCACAACAACTGACG |
| IRAK4-F | AGCTTGCAGCAATGGTTGAC |
| IRAK4-F | TGTGCCAAGAAAGTGGTGGA |
| EphA2-F | AAGACATACGTGGACCCCCA |
| EphA2-R | GCTTTCAGCGTCTTGATGGC |

# Supplementary Figures and Figure Legends

#

**Figure S1. Function of IRAK4 and EphA2 on the level of cleaved caspase-1 p20 and p-NF-κB in THP-1 cells, respectively.** THP-1 cells were transfected with negative control siRNA (si-NC), IRAK4 siRNA(si-IRAK4) or EphA2 siRNA(si-EphA2) for 48 h. **(a)** Western blot analysis of cleaved caspase-1 p20 in THP-1 cells treated with MSU treatment for 3 h after transfection with si-NC or si-IRAK4. **(b)** Western blot analysis of p-NF-κB in THP-1 cells treated with MSU treatment for 3 h after transfection with si-NC or si-EphA2. These data are representative of three experiments.

**Figure S2. miR-302b repress MSU-induced IL-1β expression.** THP-1 cells were transfected with **(a)** 302b-m, **(b)** si-IRAK4 or **(c)** si-EphA2 for 48 h. ELISA analysis of IL-1β protein levels in cell culture medium 8 h after MSU treatment. These data are representative of three experiments and are shown as means ± SEMs (***p<0.001, **p<0.01, *p<0.05 by T-test).

**Figure S3. miR-302b serum levels in gouty arthritis patients and healthy controls.** Thirty-eight serum samples of gouty arthritis patients (n=18) and healthy controls (n=20) were selected for verification of the miR-302b expression levels by real-time qPCR. The serum miR-302b levels were normalized to cel-miR-39 (spiked-in synthetic miRNA as an internal control). These data are shown as the median and means ± SEMs (***p<0.001, **p<0.01, *p<0.05 by T-test).
